# Two-year follow-up of a novel fully 3D-printed off-the-shelf humeral total shoulder arthroplasty prosthesis

**DOI:** 10.1016/j.jseint.2026.101727

**Published:** 2026-05-07

**Authors:** Jie J. Yao, Adam Lencer, Jay M. Levin, Maneesha H. Palakurthi, Andrew Jawa, J. Michael Wiater, Anand M. Murthi, Matthew J. Smith, Derek J. Cuff, Samantha Riebesell, Dennis DeBernardis, Luke S. Austin

**Affiliations:** a Department of Orthopaedic Surgery, New York University, New York, NY, USA; b Jefferson Health New Jersey, Orthopedic Residency Program, Stratford, NJ, USA; c Rothman Orthopaedic Institute, Philadelphia, PA, USA; d Department of Orthopaedic Surgery, New England Baptist Hospital, Boston, MA, USA; e Boston Sports and Shoulder Center, Waltham, MA, USA; f Beaumont Health, Royal Oak, MI, USA; g MedStar Union Memorial Hospital, Baltimore, MD, USA; h Department of Orthopaedic Surgery, University of Missouri, Columbia, MO, USA; i Suncoast Orthopaedic Surgery & Sports Medicine, Venice, FL, USA; j Pennsylvania State College of Medicine, Hershey, PA, USA

**Keywords:** Anatomic total shoulder arthroplasty, Shoulder replacement, 3D-printed, Additive manufacturing, Patient-reported outcome measures, Humeral prosthesis, Prosthetic survivorship, Radiolucent

## Abstract

**Background:**

The purpose of this study was to provide 2-year post-operative clinical outcomes and survivorship of a novel, fully 3D-printed humeral prosthesis.

**Methods:**

This is a prospective case series of 34 patients who underwent anatomic total shoulder arthroplasty (TSA) with a fully 3D-printed humeral prosthesis. Minimum post-operative follow-up was two years. Patient demographics, clinical outcomes, and radiographic outcomes were collected. X-rays were examined for radiolucent lines surrounding the implant. The primary outcome was TSA survivorship. Secondary outcomes were patient-reported outcome measure (PROM) scores and radiographic findings.

**Results:**

At a minimum follow-up of 2 years, there were no revisions or reoperations with a prosthetic survivorship of 100%. At final follow-up, patients had significant improvement in American Shoulder and Elbow Surgeons and visual analog scale scores (*P* < .001), with a mean post-operative American Shoulder and Elbow Surgeons of 93 and visual analog scale of 0.5. The rate of any periprosthetic radiolucent line on X-ray was 2 of 34 (6%). All identified radiolucent lines were <0.5 mm.

**Discussion:**

The results of early clinical follow-up of this fully 3D-printed, off-the-shelf humeral prosthetic are encouraging. Post-operative radiolucent lines appear to be minimal in thickness, infrequent, and of unclear clinical significance given 100% survivorship and reassuring PROMs. Further clinical follow-up of this and other 3D-printed systems is necessary to confirm that additive manufacturing is a mechanically durable and viable method for off-the-shelf TSA manufacturing.

Improvements in the efficiency and cost of additive manufacturing, commonly known as 3D printing, have led to increased interest in its use for medical device fabrication.^9^^,^^11^^,^^18^ Numerous total shoulder arthroplasty (TSA) prostheses are now made partially incorporating additive manufacturing, relying on conventional methods for stem production with subsequent application of a 3D-printed surface coat.^7^^,^^18^ However, a fully 3D-printed prosthesis offers numerous potential advantages, including improved pore precision, overall porosity, topology optimization, spatial variability in stiffness, communication between pores, and strut size.^2^^,^^7^^,^^18^

Due to historical cost and efficiency limitations, fully 3D-printed stems have been previously limited to custom-design applications rather than standard, off-the-shelf prosthetics. Furthermore, there are numerous theoretical concerns surrounding potential inferior mechanical performance with fully 3D-printed implants (eg potentially higher rates of fatigue, increased in vivo particle release, etc.) compared to conventionally fabricated implants.^7^^,^^18^ As the efficiency, cost, and reliability of additive manufacturing improve, implant companies may utilize 3D printing more commonly to fabricate prostheses in order to enhance osseointegration and mitigate stress shielding. To our knowledge, no reports of the clinical outcomes of fully 3D-printed, off-the-shelf TSA prostheses currently exist. Therefore, the purpose of this study was to provide a minimum of 2-year post-operative clinical outcomes and survivorship of a novel 3D-printed humeral TSA prosthesis.

## Materials and methods

### Study cohort

This study is an institutional review board–approved single-surgeon prospective case series involving 34 patients (Table I) who underwent anatomic TSA with a fully 3D-printed humeral prosthesis. Patients were followed for a minimum of two years post-operatively (mean, 25 ± 1.4 months). A priori power analysis for the primary outcome was performed with a power of 0.8 and an alpha of 0.05, which recommended a sample size of 34 patients. There were 16 males and 18 females, with a mean age of 69 ± 7 years and a mean body mass index of 31.3 ± 6 kg/m^2^. Five patients (15%) had diabetes. The primary indication for surgery was osteoarthritis (97%), with 1 patient presenting with inflammatory arthritis (3%).

**Table I:** Patient demographics and pre-operative variables.

Variable	Value∗
Male sex	16 (47%)
Age at procedure (yr)	69 ± 7
Body mass index (kg/m^2^)	31.3 ± 6
Diabetes	5 (15%)
Pre-operative indication	
Osteoarthritis	33 (97%)
Inflammatory arthritis	1 (3%)

∗ Continuous variables presented as mean ± standard deviation, categorical variables presented as frequency (percentage).

### Surgical technique

The humeral prosthesis utilized in this study was a fully 3D-printed and off-the-shelf anatomic TSA (INHANCE Shoulder System, Depuy Synthes, Raynham, MA). Eight patients (23.5%) received a short-stem humeral prosthesis, while 26 patients (76.5%) received a stemless humeral prosthesis. All surgeries were conducted through a standard deltopectoral approach. Each patient received a pre-operative interscalene nerve block. All patients on visual intraoperative examination had intact rotator cuff tendons. The subscapularis was managed with a lesser tuberosity osteotomy. After final implantation of the TSA, the lesser tuberosity osteotomy was repaired using sutures passed through lateral bone tunnels. Corresponding glenoid components used in these patients were circular, highly cross-linked, zone-conforming, all-polyethylene, and cemented with a recessed technique. All patients received a layered closure with absorbable suture, glue, and a sterile dressing.

### Outcomes

**Figure 1:**
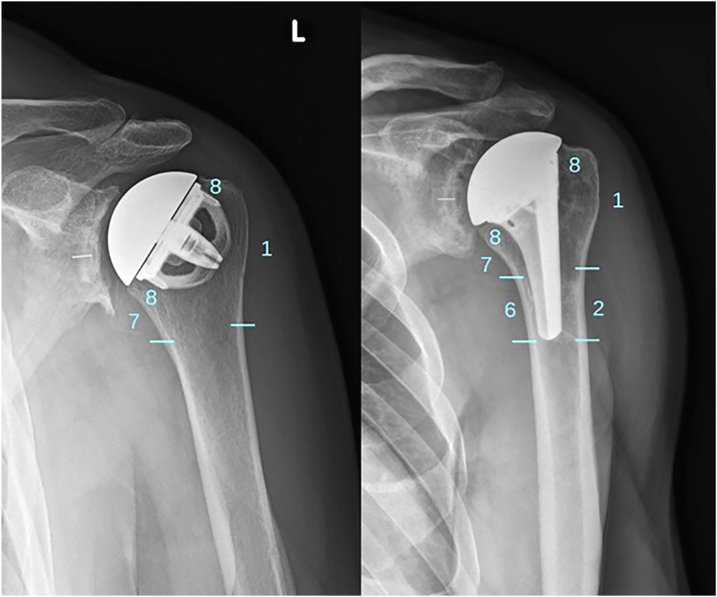
Radiographic zones of post-operative radiolucent lines. *Left*: stemless implants impact only the (1) greater tuberosity, (7) medial calcar, and (8) collar regions. *Right*: short-stem implants also reach the (2) lateral metadiaphyseal and (6) medial metadiaphyseal zones.

The primary outcome was TSA survivorship free from revision. Secondary outcomes included changes in patient-reported outcome measures (PROMs) and the presence of radiolucent lines on post-operative X-rays. The PROMs included American Shoulder and Elbow Surgeons (ASES), Single Assessment Numeric Evaluation (SANE), and visual analog scale (VAS) shoulder pain scores.^3^^,^^8^^,^^16^ Radiolucent lines were graded with a modification to previously described methods.^13^^,^^14^ The thickness of the lines was categorized as < 0.5 mm, <1.0 mm, <1.5 mm, and <2.0 mm, and the locations of radiolucent lines were also recorded (Fig. 1).

### Data collection

Pre-operative and post-operative PROMs were collected, including ASES and VAS scores at a minimum 2-year follow-up. Post-operative X-rays at a minimum of two years were evaluated by 3 independent shoulder fellowship-trained orthopedic surgeons (JY, LA, and DD) for the presence and thickness of radiolucent lines in selected zones of lucency. The same 3 reviewers then evaluated these same X-rays two weeks later. From the 6 readings for each zone of lucency, the median value was used as the final datapoint.

### Statistical analysis

Data were analyzed using descriptive statistics, including frequencies (percentages) and mean ± standard deviation. Cohen kappa coefficient was used to assess inter-rater and intra-rater reliability for radiographic lucency grading, with values 0-0.2 considered no agreement, 0.2-0.4 minimal agreement, 0.41-0.60 fair agreement, 0.61-0.80 moderate agreement, 0.81-0.99 excellent agreement, and 1.00 perfect agreement. In addition, pre-operative PROMs were compared to post-operative PROMs to assess for statistically significant improvement in shoulder pain and function using a paired samples *t*-test (Microsoft Excel Version 2407; MIcrosoft Corp., Redmond, WA, USA). A *P* value <.05 was considered significant.

## Results

At a minimum follow-up of 2 years, there were no revisions or reoperations, with a prosthetic survivorship of 100%. Significant improvements in PROMs were observed, with ASES scores increasing from a mean of 36.8 ± 15.7 to 93.1 ± 8.4 (*P* < .001) and VAS pain scores decreasing from 7.4 ± 1.4 to 0.5 ± 0.8 (*P* < .001). The mean SANE score at two years was 91.3 ± 11.6 (Table II). The rate of any periprosthetic radiolucent line on XR was 2 of 34 (5.9%). Interobserver reliability ranged from none (0.015 to 1.000) to perfect, and intraobserver reliability ranged from none to perfect (0.015-1.000) (Table III). All identified radiolucent lines were <0.5 mm. There were no prosthetics with radiolucent lines in the medial calcar or medial metadiaphyseal zones.

**Table II:** Two-year patient outcomes.

Variable	Pre-operative value∗	Post-operative value∗	Change∗	*P* value
Revision of components		0 (0%)		
Reoperation		0 (0%)		
Patient-reported outcome measures				
SANE (%)	N/A	91.3% ± 11.6		
ASES	36.8 ± 15.7	93.1 ± 8.4	56.6 ± 18.8	<.001§
VAS (mm)	7.4 ± 1.4	0.5 ± 0.8	−6.9 ± 1.4	<.001§
Humeral radiolucent lines frequency by region†‡				
Greater tuberosity		1 of 34		
Lateral metadiaphyseal†		1 of 8		
Medial metadiaphyseal†		0 of 8		
Medial calcar		0 of 34		
Collar		1 of 34		

*SANE*, Single Assessment Numeric Evaluation; *ASES*, American Shoulder and Elbow Surgeons; *VAS*, visual analog scale.

∗ Continuous variables presented as mean ± standard deviation, categorical variables presented as frequency (percentage).

† Stemless components do not reach the metadiaphyseal region; therefore, the percentages for these regions are calculated only considering stemmed components. (Fig. 1).

‡ All radiolucent lines were <0.5 mm in thickness.

§ *P* values's <.05 were considered significant.

**Table III:** Inter- and intrarater reliability of radiolucency measurements.

Variable	Cohen kappa	Agreement
Total data between all readers		
Zone 1	0.042	None
Zone 2	0.207	Minimal
Zone 6	1.000	Perfect
Zone 7	0.057	None
Zone 8	0.129	None
First reading between all readers		
Zone 1	0.100	None
Zone 2	0.111	None
Zone 6	1.000	Perfect
Zone 7	0.015	None
Zone 8	0.024	None
Second reading between all readers		
Zone 1	0.059	None
Zone 2	0.238	Minimal
Zone 6	1.000	Perfect
Zone 7	0.046	None
Zone 8	0.016	None
Reviewer 1 only		
Zone 1	0.640	Moderate
Zone 2	0.590	Moderate
Zone 6	1.000	Perfect
Zone 7	1.000	Perfect
Zone 8	1.000	Perfect
Reviewer 2 only		
Zone 1	0.380	Minimal
Zone 2	0.333	Minimal
Zone 6	1.000	Perfect
Zone 7	0.372	Minimal
Zone 8	0.162	None
Reviewer 3 only		
Zone 1	0.015	None
Zone 2	1.000	Perfect
Zone 6	1.000	Perfect
Zone 7	1.000	Perfect
Zone 8	0.015	None

## Discussion

The results of this study demonstrate promising early clinical outcomes for a fully 3D-printed off-the-shelf humeral prosthesis in anatomic TSA. The primary outcome of interest was TSA survivorship. No patients in this series had undergone a revision TSA or reoperation at 2 years post-operative follow-up. This is consistent with the high short-term survivorship of conventionally fabricated anatomic TSA stems, which has been estimated at 99%-100% at 2 years.^1^^,^^6^ Our early case series suggests that this fully 3D-printed humeral stem has noninferior survivorship compared to conventionally fabricated TSA stems as reported in the literature. Secondary outcomes of this study included PROMs and radiographic findings at short-term follow-up. The noted improvement in PROMs in our study was consistent with previously published accounts of changes in PROMs after TSA with conventionally fabricated stems.^5^^,^^15^ A select few patients demonstrated minor radiolucent lines evident on the lateral, tension side of the humeral component on post-operative X-ray. Radiolucent lines around the humeral stem have traditionally been considered a radiographic indicator of potential loosening and failure.^13^^,^^14^ However, the clinical significance of these lines in our case is unclear, as they were not associated with progressive loosening, osteolysis, or reoperation. While these initial results are encouraging, close longer-term follow-up is necessary to fully assess the durability and clinical performance of fully 3D-printed humeral prostheses.

Our findings align with existing literature on fully 3D-printed implants in the fields of total hip arthroplasty and spinal fusion surgery. In total hip arthroplasty, completely 3D-printed acetabular cups have had noninferior 10-year all-cause revision free survivorship compared to conventionally manufactured acetabular components.^4^ Furthermore, radiographic evaluation of novel 3D-printed porous interbody cages used in lateral lumbar interbody fusion demonstrated improved posterior fusion rates and reduced subsidence compared to conventional threaded cages, with comparable PROMs.^17^ There is a small but growing body of literature demonstrating noninferior survivorship of completely 3D-printed implants in vivo when compared to similar conventionally fabricated implants.

There are limitations inherent to this study. Firstly, the follow-up is short-term for arthroplasty, and it remains to be seen if long- or mid-term follow-up would reveal less-promising findings. Conventionally fabricated anatomic TSA stems have been shown in recent studies to have a long-term survivorship of 96% at 10 years.^10^^,^^12^ However, early reports of novel prostheses and manufacturing methods are still valuable to demonstrate the early promise of this new technology. Secondly, this study was designed without a control group. While a cohort of control patients undergoing a TSA with a conventionally fabricated prosthesis would have been an option, different TSA systems do not have equivalent instrumentation and prosthetic geometry, making it difficult to precisely investigate differences between various implant groups. Nevertheless, the outcomes from this cohort compare favorably to historical data regarding TSA outcomes with conventionally fabricated prostheses. Third, all reported radiolucent lines within this study were <0.5 mm in width, thus making them difficult to consistently appreciate on plain radiographs and the most likely reason for our interobserve reliability range of 0.015-1. Lastly, this study is a small cohort study of only 34 patients with a single high-volume TSA surgeon- which limits the statistical power that our cohort may have to detect rare complications or subtle differences between groups. It is unknown whether these findings would be replicated in a larger group of patients across multiple surgeons.

## Future considerations

Future prospective randomized studies with larger cohorts and matched comparisons to conventional implants are warranted to better assess outcomes. Inclusion of multiple surgeons would enhance generalizability and improve follow up, helping to confirm whether fully 3D-printed, off-the-shelf prostheses demonstrate noninferior survivorship and clinical outcomes. In addition, dedicated analyses could evaluate the learning curve associated with adoption of 3D-printed, off-the-shelf implants, as well as their overall cost implications.

## Conclusion

A novel fully 3D-printed, off-the-shelf humeral prosthesis used in anatomic TSA demonstrates excellent short-term clinical outcomes, with significant improvements in PROMs and a 100% survivorship rate at two years.

## Disclaimers:

Funding: No funding was disclosed by the authors.

Conflicts of interest: Jay M. Levin owns stock or stock options in Stryker and Zimmer. Andrew Jawa serves as a board or committee member of AAOS, American Shoulder and Elbow Surgeons; receives other financial or material support from Boston Outpatient Surgical Suites, DePuy; is a paid consultant, paid presenter, or speaker for DJ Orthopedics; receives IP royalties and holds stock/stock options from Ignite Orthopedics; is on the editorial or governing board of the Journal of Shoulder and Elbow Surgery; and receives publishing royalties, financial, or material support from Oberd. J. Michael Wiater serves as a board or committee member of AAOS, American Shoulder and Elbow Surgeons; receives IP royalties from DePuy, Ignite Orthopedics, Innomed, Smith & Nephew; owns stock or stock options in Catalyst OrthoScience LLC, Coracoid Solutions LLC, Ignite Orthopedics; is a paid consultant and presenter or speaker for DePuy; receives research support from Zimmer, and serves on the Editorial or governing board of Seminars in Arthroplasty: JSES.

Anand M. Murthi serves as a board or committee member of the AAOS, American Shoulder and Elbow Surgeons, Association of Clinical Elbow and Shoulder Surgeons, MidAtlantic Shoulder and Elbow Society; serves on the editorial or governing board of Current Orthopedic Practice, Journal of Bone and Joint Surgery, Journal of Shoulder and Elbow Arthroplasty, Journal of Shoulder and Elbow Surgery; has IP royalties from Ignite Orthopedics, Depuy Inc, and Globus Medical; does consulting for Ignite Orthopedics, Depuy Inc, Globus Medical, Work Rehab Solutions, Aevumed, vTail, and Zimmer; owns stock in Catalyst Orthoscience, Aevumed, Replicare, Restore3D, vTail, and Ignite Orthopedics; receives publishing royalties and other support from Wolters Kluwer Health - Lippincott Williams & Wilkins and Current Orthopedic Practice; and receives research support from Zimmer and Stryker.

Matthew J. Smith receives IP royalties and is a paid consultant and presenter or speaker for DePuy; receives IP royalties and stock or stock options from Ignite Orthopedics; serves as a board or committee member of American Shoulder and Elbow Surgeons; serves on the editorial or governing board of Current Orthopedic Practice.

Derek J. Cuff receives IP royalties from, is a paid consultant for, and paid presenter or speaker for both DePuy and Ignite Orthopedics; owns stock or stock options in Ignite Orthopedics.

Luke Austin receives IP royalties and is a paid consultant for DePuy and Ignite Orthopedics; has stock or stock options in Ignite Orthopedics; has received other financial or material support from Rothman Institute and Related Holdings; and has received research support from Zimmer.

Any additional authors, their immediate families, and any research foundations with which they are affiliated have not received any financial payments or other benefits from any commercial entity related to the subject of this article.

## References

[1] Aldinger P.R., Raiss P., Rickert M., Loew M. (2010). Complications in shoulder arthroplasty: an analysis of 485 cases. Int Orthop (SICOT).

[2] Bagaria V., Bhansali R., Pawar P. (2018). 3D printing- creating a blueprint for the future of orthopedics: current concept review and the road ahead. J Clin Orthop Trauma.

[3] Boonstra A.M., Schiphorst Preuper H.R., Reneman M.F., Posthumus J.B., Stewart R.E. (2008). Reliability and validity of the visual analogue scale for disability in patients with chronic musculoskeletal pain. Int J Rehabil Res.

[4] Castagnini F., Bordini B., Cosentino M., Pardo F., Gorgone M., Traina F. (2024). Modern cementless acetabular cups in total hip arthroplasty performed for primary osteoarthritis: a comparative registry study. Arch Orthop Trauma Surg.

[5] Davies A.R., Sabharwal S., Liddle A.D., Zamora-Talaya B., Rangan A., Reilly P. (2024). Patient-reported outcomes following total shoulder arthroplasty and hemiarthroplasty: an analysis of data from the National Joint Registry. J Shoulder Elbow Surg.

[6] Deshmukh A.V., Koris M., Zurakowski D., Thornhill T.S. (2005). Total shoulder arthroplasty: Long-term survivorship, functional outcome, and quality of life. J Shoulder Elbow Surg.

[7] Martelli N., Serrano C., Van Den Brink H., Pineau J., Prognon P., Borget I. (2016). Advantages and disadvantages of 3-dimensional printing in surgery: a systematic review. Surgery.

[8] Michener L.A., McClure P.W., Sennett B.J. (2002). American Shoulder and Elbow Surgeons Standardized Shoulder Assessment Form, patient self-report section: reliability, validity, and responsiveness. J Shoulder Elbow Surg.

[9] Mishra A., Srivastava V. (2021). Biomaterials and 3D printing techniques used in the medical field. J Med Eng Technol.

[10] Piper C., Neviaser A. (2022). Survivorship of anatomic total shoulder arthroplasty. J Am Acad Orthop Surg.

[11] Popov V.V., Muller-Kamskii G., Kovalevsky A., Dzhenzhera G., Strokin E., Kolomiets A. (2018). Design and 3D-printing of titanium bone implants: brief review of approach and clinical cases. Biomed Eng Lett.

[12] Rasmussen J.V., Hole R., Metlie T., Brorson S., Äärimaa V., Demir Y. (2018). Anatomical total shoulder arthroplasty used for glenohumeral osteoarthritis has higher survival rates than hemiarthroplasty: a Nordic registry-based study. Osteoarthritis Cartilage.

[13] Sanchez-Sotelo J., Wright T.W., O’Driscoll S.W., Cofield R.H., Rowland C.M. (2001). Radiographic assessment of uncemented humeral components in total shoulder arthroplasty. J Arthroplasty.

[14] Sperling J.W., Cofield R.H., O’Driscoll S.W., Torchia M.E., Rowland C.M. (2000). Radiographic assessment of ingrowth total shoulder arthroplasty. J Shoulder Elbow Surg.

[15] Su F., Nuthalapati P., Feeley B.T., Lansdown D.A. (2023). Outcomes of anatomic and reverse total shoulder arthroplasty in patients over the age of 70: a systematic review. JSES Rev Rep Tech.

[16] Thigpen C.A., Shanley E., Momaya A.M., Kissenberth M.J., Tolan S.J., Tokish J.M. (2018). Validity and responsiveness of the single alpha-numeric evaluation for shoulder patients. Am J Sports Med.

[17] Velluto C., Mundis G., Scaramuzzo L., Perna A., Capece G., Cruciani A. (2024). Radiological evaluation of fusion patterns after Lateral Lumbar Interbody fusion with 3D-printed porous titanium cages vs. conventional titanium cages. Front Surg.

[18] Wixted C.M., Peterson J.R., Kadakia R.J., Adams S.B. (2021). Three-dimensional printing in orthopaedic surgery: current applications and future developments. JAAOS Glob Res Rev.

